# Spontaneous regression of a giant hepatic hemangioma on serial MRI: A case report with 3-year follow-up

**DOI:** 10.1016/j.radcr.2026.06.162

**Published:** 2026-07-25

**Authors:** Denis Qirinxhi, Durim Çela, Blerina Saraci, Fatmir Bilaj

**Affiliations:** a Department of Radiology, University Hospital Center “Mother Teresa”, Tirana, Albania; b Faculty of Medicine, University of Tirana, Tirana, Albania; c Department of Radiology, Boston Medical Center, Boston, MA, USA; d Chobanian & Avedisian School of Medicine, Boston University, Boston, MA, USA

**Keywords:** Hepatic hemangioma, Spontaneous regression, Magnetic resonance imaging, Liver lesion, Cavernous hemangioma, Conservative management

## Abstract

Hepatic hemangiomas are the most common benign mesenchymal tumors of the liver and usually demonstrate stable imaging characteristics over time, whereas spontaneous regression in adults remains uncommon and poorly documented in literature. We report the case of a 53-year-old woman with a giant hepatic hemangioma incidentally detected on magnetic resonance imaging (MRI) during evaluation of chronic nonspecific abdominal discomfort. Serial MRI examinations performed in February 2022, August 2023, and January 2025 demonstrated classic imaging findings of cavernous hemangioma, including marked T2 hyperintensity and peripheral nodular discontinuous enhancement with progressive centripetal fill-in, followed by substantial spontaneous regression without medical, surgical, or interventional treatment. Lesion dimensions changed from 14.1 × 9.4 × 9.5 cm to 15.9 × 10.0 × 9.5 cm, followed by regression to 8.0 × 5.4 × 4.7 cm, corresponding to an estimated volumetric reduction exceeding 80%. The patient remained clinically stable throughout follow-up, with no evidence of malignant transformation or clinically significant laboratory abnormalities. This case highlights that giant hepatic hemangiomas with characteristic MRI features may demonstrate substantial spontaneous regression during long-term follow-up and supports conservative management in clinically stable patients with typical imaging findings.

## Introduction

Hepatic hemangiomas (HHs) are the most common benign tumors of the liver, with reported prevalence ranging from 0.4% to 20% depending on imaging modality and autopsy series [1,2]. They occur more frequently in women and are often discovered incidentally during abdominal imaging performed for unrelated indications [1]. Histologically, most lesions correspond to cavernous hemangiomas composed of dilated vascular channels lined by endothelial cells and separated by fibrous septa [2].

The majority of hepatic hemangiomas remain asymptomatic and stable in size over time. Lesions larger than 4-5 cm are commonly referred to as giant hemangiomas and may occasionally produce nonspecific symptoms, including abdominal discomfort, fullness, early satiety, or rarely complications such as rupture or consumptive coagulopathy [3,4]. In most cases, conservative management with imaging follow-up is recommended when imaging findings are characteristic [5].

Magnetic resonance imaging (MRI) plays a central role in diagnosis because of its high sensitivity and specificity. Typical MRI findings include hypointensity on T1-weighted images, marked hyperintensity on T2-weighted images, and peripheral nodular discontinuous enhancement with progressive centripetal fill-in following gadolinium administration [6]. These imaging characteristics usually allow confident noninvasive diagnosis.

The natural history of hepatic hemangiomas is generally characterized by stability or slow growth. Spontaneous regression in adults has only rarely been documented and remains incompletely understood [7,8]. Proposed mechanisms include intralesional thrombosis, progressive fibrosis, hyalinization, and vascular involution [8,9].

We present a case of a giant hepatic hemangioma demonstrating substantial spontaneous regression documented on serial MRI examinations over a 3-year period.

## Case presentation

A 53-year-old woman underwent abdominal MRI for evaluation of chronic nonspecific abdominal discomfort. Her medical history was notable only for erosive gastritis previously diagnosed by upper gastrointestinal endoscopy. She had no history of chronic liver disease, viral hepatitis, malignancy, thromboembolic disease, vascular malformations, or previous hepatic pathology. The patient had no prior abdominal surgery. Family history was unremarkable for liver disease, vascular disorders, or malignancy. No previous abdominal imaging studies were available for comparison at the time of diagnosis. MRI revealed a large, well-circumscribed lesion occupying the right hepatic lobe with imaging characteristics typical of cavernous hepatic hemangioma.

**Fig. 1:**
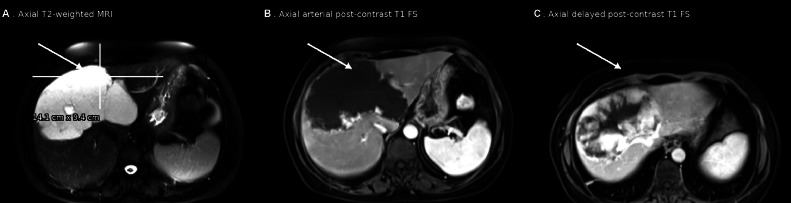
Baseline MRI examination (February 2022). (A) Axial T2-weighted image demonstrates a markedly hyperintense giant hepatic hemangioma; white arrows indicate the lesion and measurement lines show transverse and anteroposterior dimensions of 14.1 × 9.4 cm. (B) Axial arterial-phase post-contrast T1-weighted fat-suppressed image demonstrates peripheral nodular discontinuous enhancement. (C) Axial delayed postcontrast T1-weighted image demonstrates progressive centripetal fill-in characteristic of hepatic hemangioma.

Initial MRI performed in February 2022 demonstrated the typical imaging appearance of a giant hepatic hemangioma (Fig. 1):

• Well-defined hepatic lesion with lobulated margins.
• Hypointensity on T1-weighted images.
• Marked homogeneous hyperintensity on T2-weighted images.
• Peripheral nodular discontinuous enhancement during the arterial phase.
• Progressive centripetal fill-in on delayed postcontrast sequences.

No imaging evidence of chronic liver disease, cirrhosis, portal hypertension, or additional focal hepatic lesions was identified.

The patient reported intermittent abdominal discomfort and had previously been evaluated by a gastroenterologist, who diagnosed erosive gastritis. The symptoms were considered nonspecific and could not be confidently attributed to the hepatic hemangioma. No hepatology or surgical consultation was performed because imaging findings were considered characteristic of benign cavernous hemangioma and the patient remained clinically stable throughout follow-up.

No surgical, interventional, or pharmacological treatment directed toward the hemangioma was administered during follow-up. Specifically, the patient did not receive antiangiogenic therapy, chemotherapy, corticosteroids, or beta-blocker treatment, which have been reported in the literature to potentially influence hemangioma behavior. The patient also reported no history of oral contraceptive or hormonal therapy use.

**Fig. 2:**
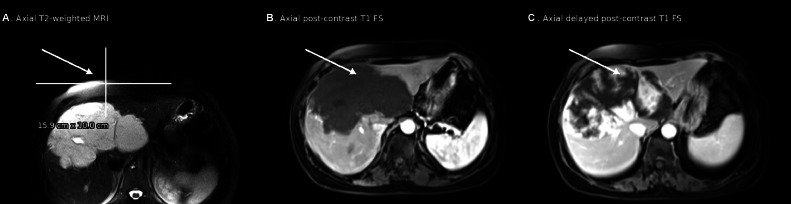
Follow-up MRI examination (August 2023). (A) Axial T2-weighted image demonstrates mild interval enlargement while preserving typical T2 hyperintensity; white arrows indicate the lesion and measurement lines show transverse and anteroposterior dimensions of 15.9 × 10.0 cm. (B) Axial postcontrast T1-weighted image again demonstrates peripheral nodular enhancement. (C) Axial delayed post-contrast T1-weighted image demonstrates persistent progressive centripetal enhancement without suspicious washout.

**Fig. 3:**
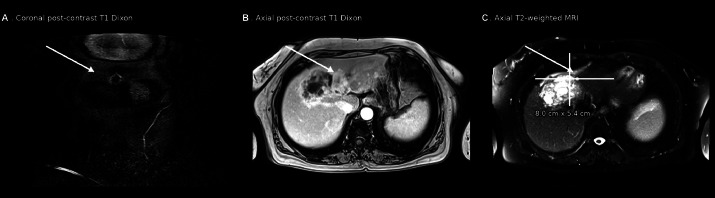
Final MRI examination (January 2025). (A) Coronal postcontrast T1-weighted image demonstrates marked interval reduction in lesion volume. (B) Axial postcontrast T1-weighted image demonstrates persistent benign enhancement characteristics. (C) Axial T2-weighted image confirms substantial spontaneous regression; white arrows indicate the residual lesion and measurement lines show transverse and anteroposterior dimensions of 8.0 × 5.4 cm.

Serial MRI examinations performed in February 2022, August 2023, and January 2025 demonstrated an initial mild increase in lesion dimensions followed by marked spontaneous regression. The serial MRI findings are illustrated in Fig. 1, Fig. 2, Fig. 3.

Lesion measurements were as follows:

**Table:** 

Examination date	Transverse (cm)	Anteroposterior (cm)	Longitudinal (cm)
February 2022	14.1	9.4	9.5 (not shown in figures)
August 2023	15.9	10.0	9.5 (not shown in figures)
January 2025	8.0	5.4	4.7 (not shown in figures)

Overall, the lesion demonstrated greater than 50% reduction in maximal lesion diameter relative to peak size, corresponding to an estimated volumetric reduction exceeding 80%.

Routine laboratory investigations performed during follow-up remained within normal limits. Liver function tests showed normal ALT, AST, ALP, GGT, and bilirubin levels. Complete blood count and coagulation parameters, including platelet count and INR, were also within reference ranges. No laboratory evidence of consumptive coagulopathy, liver dysfunction, or systemic inflammatory disease was identified during follow-up.

At the most recent follow-up examination in January 2025, the patient remained clinically stable and asymptomatic, with no evidence of liver dysfunction, hemorrhage, Kasabach–Merritt syndrome, malignant transformation, or other hemangioma-related complications.

Follow-up MRI examinations continued to demonstrate imaging characteristics typical of benign hemangioma (Fig. 2, Fig. 3).

## Discussion

Hepatic hemangiomas are commonly encountered benign vascular liver lesions that are typically managed conservatively because of their benign biological behavior and characteristic imaging appearance [1,5,10,11]. Most lesions remain stable over time, although interval growth may occur, particularly in larger lesions and in female patients [3]. In contrast, spontaneous regression in adults is distinctly uncommon and remains poorly understood.

Several mechanisms have been proposed to explain spontaneous involution of hepatic hemangiomas. Histopathological studies suggest that fibrosis, sclerosis, vascular obliteration, and progressive hyalinization may contribute to lesion regression [8,9,12].

In the present case, serial MRI examinations demonstrated preservation of characteristic benign imaging findings throughout the follow-up period despite substantial reduction in lesion size. The lesion consistently demonstrated marked T2 hyperintensity and progressive centripetal enhancement without imaging features suggestive of malignancy.

The differential diagnosis of atypical or changing vascular hepatic lesions includes hepatic angiosarcoma, inflammatory lesions, treated metastases, and atypical hepatocellular carcinoma. However, the persistent classic enhancement pattern observed in this case strongly supported the diagnosis of benign cavernous hemangioma [6,13]. Furthermore, the prolonged imaging follow-up demonstrated progressive involution rather than aggressive biological behavior.

Previous reports describing spontaneous regression of hepatic hemangiomas remain limited and consist predominantly of isolated case reports or small series [7,8,14]. Onishi et al. [7] reported that giant hepatic hemangiomas may demonstrate variable longitudinal imaging behavior, whereas Zhang et al. [9] described substantial regression without surgical treatment in a clinically stable patient [7,9]. Some studies have suggested that regression may occur more commonly in older patients due to progressive fibrosis and decreased vascularity [8]. Hormonal and vascular factors have also been proposed, although definitive mechanisms remain uncertain.

No documented laboratory or imaging findings suggested consumptive coagulopathy, malignant transformation, or other secondary pathological process during follow-up.

The uniqueness of the present case lies in the substantial spontaneous regression of a giant hepatic hemangioma documented by serial MRI examinations over a 3-year period without any form of treatment. Although spontaneous involution has been reported previously, detailed longitudinal MRI documentation remains relatively uncommon in literature. The preservation of classic benign enhancement characteristics throughout follow-up further strengthens diagnostic confidence and provides valuable evidence regarding the potential natural history of these lesions.

This case highlights several clinically relevant considerations. First, substantial spontaneous regression of giant hepatic hemangiomas can occur without intervention, although this phenomenon appears to be uncommon. Second, stable benign MRI characteristics remain essential for maintaining diagnostic confidence during long-term follow-up. Finally, recognition of this possible natural evolution may help prevent unnecessary biopsy, surgery, or aggressive treatment in patients with characteristic imaging findings and stable clinical status, consistent with contemporary guideline recommendations for benign focal liver lesions [10,11].

The principal limitation of this report is its single-case design, which limits generalizability. Nevertheless, the long-term MRI follow-up provides valuable imaging documentation of spontaneous involution.

## Conclusion

Spontaneous regression of giant hepatic hemangiomas is rare but may occur during long-term follow-up. In lesions demonstrating characteristic MRI findings and stable benign imaging behavior, conservative management with periodic imaging surveillance remains an appropriate strategy in clinically stable patients with characteristic imaging findings. Recognition of this potential natural evolution may help avoid unnecessary invasive diagnostic or therapeutic procedures.

## Patient consent

Written informed consent was obtained from the patient for publication of this case report and accompanying images. A copy of the written consent is available for review by the Editor-in-Chief of this journal on request.
